# Age-related changes in foot kinematics during walking

**DOI:** 10.1186/1757-1146-7-S1-A4

**Published:** 2014-04-08

**Authors:** John Arnold, Shylie Mackintosh, Sara Jones, Dominic Thewlis

**Affiliations:** 1 Biomechanics and Neuromotor Laboratory, School of Health Sciences, University of South Australia, Adelaide, Australia; 2 Sansom Institute for Health Research, University of South Australia, Adelaide, Australia; 3 International Centre for Allied Health Evidence (iCAHE), University of South Australia, Adelaide, Australia

## Background

Differences in dynamic foot function between young and older adults have been reported [1], however foot kinematics during walking remain largely unquantified. Our understanding of foot kinematics during walking is largely based on single-segment foot models, which limits the inferences that can be made about foot motion. This study aimed to determine if differences in foot kinematics existed between young and older adults during walking using a multi-segment foot model [2].

## Participants and methods

Forty adults participated– 20 young adults (10F:10M, mean age 23.2 years SD 3.0, height 1.75 m SD 0.1, mass 73.6 kg SD 19.5) and 20 older adults (11F:9M, mean age 73.2 years, height 1.69 m SD 0.11, mass 76.9 kg SD 15.5). Surface markers were attached to anatomical landmarks consistent with the protocol by Leardini et al [2]. Kinematic and kinetic data were acquired with 12 cameras (VICON MX-F20, 100 Hz) and two Kistler force platforms (9281B, 400 Hz). Five walking trials were obtained for both groups at a self-selected speed with an additional five trials from the young adults at a slow speed. Joint angles were computed using the joint coordinate system [3]. Variables of interest were the joint angles between the calcaneus-shank, midfoot-calcaneus, metatarsus-midfoot and hallux-metatarsus at initial contact, end of loading response and toe-off and the joint range of motion (ROM). Differences in means of variables between the young adults (preferred and slow speeds) and older adults were compared using Student’s t-tests. Effect sizes (Cohen’s *d*) for the differences were also computed.

## Results

**Figure 1:**
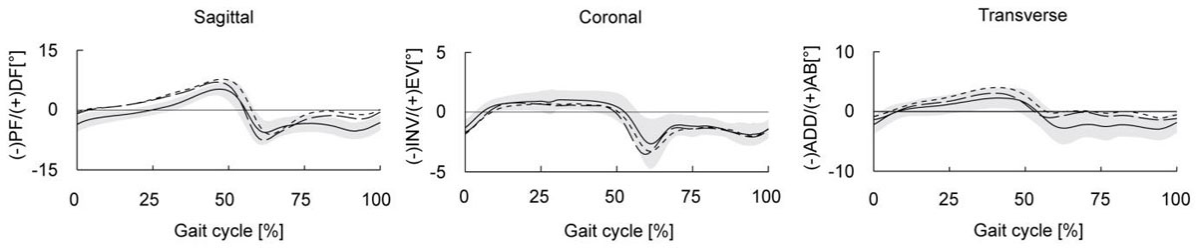
Midfoot angles for older (solid line), young (dashed) and young (slow) groups (dotted)

The older adults had a less plantarflexed calcaneus at toe-off (-9.6° vs. -16.1°, *d* = 1.0, p = <0.001), a smaller sagittal plane ROM of the midfoot (11.9° vs. 14.8°, *d* = 1.3, p = <0.001, fig. 1) and smaller coronal plane ROM of the metatarsus (3.2° vs. 4.3°, *d* = 1.1, p = 0.006) compared to the young adults. Walking speed did not alter these changes as they existed when groups walked at comparable speeds.

## Conclusions

Independent of walking speed, older adults exhibit differences in foot kinematics compared to younger adults. These are characterised by reduced mobility of the calcaneus, midfoot and metatarsus and changes in angular position of the hindfoot at toe-off. Further research is required to establish possible links to the development of pathology and their influence on broader physical function in older adults.
